# Community Nurses' Experiences Assessing Early‐Stage Pressure Injuries in People With Dark Skin Tones: A Qualitative Descriptive Analysis

**DOI:** 10.1111/jan.16533

**Published:** 2024-10-14

**Authors:** Oozageer Gunowa Neesha, Oti Adomako Kwame, Jackson Debra

**Affiliations:** ^1^ The University of Surrey Guildford England; ^2^ The University of Sydney Sydney Australia

**Keywords:** community care, district nursing, diversity and inclusion in healthcare, health disparities, health equity, inequalities in health, patient safety, pressure injury, pressure ulcers, quality of life

## Abstract

**Aim:**

To examine community nurses' experiences of caring for people with dark skin tones at high risk of developing a pressure injury.

**Design:**

Qualitative descriptive design.

**Methods:**

Focus groups and individual semi‐structured interviews were conducted among registered nurses working in the community between November 2023 and March 2024. Thematic analysis was used.

**Results:**

The findings reveal the lack of nurse education on diverse skin tones, how community nurses gain knowledge on skin tone diversity in the context of pressure injuries and the topics community nurses believe are crucial to improve the management of pressure injuries in patients with dark skin tones.

**Conclusion:**

The study highlights the gap in nurse education regarding diverse skin tones, revealing how community nurses acquire knowledge related to pressure injuries in patients with dark skin tones.

**Implications for the Profession and/or Patient Care:**

This research could inform the development of targeted educational programmes and training initiatives, ultimately preventing patient harm and enhancing the quality of care and health outcomes for patients with dark skin tones.

**Impact:**

It provides valuable insights into key topics that community nurses consider essential for improving the early recognition and management of pressure injuries in people with darker skin tones.

**Reporting Method:**

The research adhered to the Consolidated Criteria for Reporting Qualitative Research (COREQ) guidelines.

**Public Contribution:**

A project steering group contributed to the concept of the study and checked the interview questions were relevant and suitable.

**Summary**

- **What does this paper contribute to the wider global clinical community?**
  ○ The need to understand that early signs of pressure damage may be missed among people with dark skin tones.
  ○ Nurses should ask individuals at risk of pressure injuries (and their carers) about their normal skin tone and any noticeable changes to ensure accurate and early detection of pressure injuries.

## 1 Introduction

Pressure injuries cause significant debilitation and human suffering and have considerable financial and resource implications on the healthcare sector and rates continue to rise (Jackson et al. 2017; McEvoy et al. 2021; Pittman et al. 2024). Literature reveals that pressure injuries present significant emotional and psychological, physiological (manifesting as pain and discomfort) and social consequences (social isolation) on individuals (Jackson et al. 2018; Ibeh and Hambridge 2024). Providing care and support to patients with pressure injuries was estimated to cost the British National Health Service (NHS) between £1.4 and £2.1 billion annually (NHS Improvement 2018) while in the United States, the annual costs associated with treating hospital‐acquired pressure injuries alone have been estimated at more than USD27 billion (Padula and Delarmente 2019).

While patients face significant effects due to pressure injuries, it is essential to recognise that nurses are not exempt from experiencing the effects of pressure injuries. A thematic analysis of interviews with nurses caring for patients with pressure injuries revealed a spectrum of emotions including guilt, resentment, accomplishment and helplessness (Na, Yoo, and Kweon 2024). However, there is limited research exploring the experiences of community nurses managing pressure injuries across different skin tones, particularly in populations with dark skin tones, who are at a higher risk of developing severe pressure injuries (Oozageer Gunowa et al. 2018).

## 2 Background

A pressure injury arises from the combination of compression or pressure along with shear forces, resulting in damage to the skin or underlying tissue. These injuries commonly occur over bony prominences or in association with medical devices (European Pressure Ulcer Advisory Panel, National Pressure Injury Advisory Panel and Pan Pacific Pressure Injury Alliance (EPUAP/NPIAP/PPPIA) 2019). Pressure injuries have four recognised stages arranged in order of severity from 1 to 4, there are also other types, and these are as follows: unstageable pressure injury, deep tissue pressure injury, medical device‐related pressure injury and mucosal membrane pressure injury (EPUAP/NPIAP/PPPIA 2019). Early‐stage pressure injuries, known as Stage I, typically present with intact skin showing what is described as ‘non‐blanchable erythema’.

A key issue in managing and preventing pressure injuries is the early detection of ‘non‐blanchable erythema’ seen in Stage 1. Identifying erythema in people with dark skin tone poses a challenge, hindering the immediate recognition of these injuries (EPUAP/NPIAP/PPPIA 2019). The challenge in the early recognition of erythema in populations with dark skin tones could be attributed to the varied hues erythema can present in this demographic namely: deep brown, ashy grey or with a violet shade (Sangha 2021; Oozageer Gunowa, Oti, and Jackson 2024). The challenge in recognising erythema in populations with dark skin tones is further compounded by underrepresentation in the medical literature (Adelekun, Onyekaba, and Lipoff 2021; Dzuali et al. 2020; Perlman et al. 2021), in research designs (Oozageer Gunowa et al. 2024) and nurse education (Oozageer Gunowa et al. 2021). Because of difficulties associated with identifying pressure injuries at Stage 1, pressure damage is often identified at more advanced stages in people with dark skin tones (Oozageer Gunowa et al. 2018; Osborne Chambers and Thompson 2024).

With nurses assuming an important role in the prevention of pressure injuries, studies have explored the experiences of nurses in relation to managing and preventing pressure injuries. Existing studies have explored the experiences, knowledge and attitudes of nurses in pressure injury prevention and management; however, none of the studies focuses on pressure injuries in populations with dark skin tones (Athlin et al. 2010; Barakat‐Johnson et al. 2019; Beeckman et al. 2011; Dilie and Mengistu 2015; Källman and Suserud 2009; Moore and Price 2004; Mwebaza et al. 2014; Nuru et al. 2015; Tubaishat, Aljezawi, and Al Qadire 2013). While the studies by Dilie and Mengistu (2015); Mwebaza et al. (2014); and Nuru et al. (2015) were conducted in African countries (Ethiopia and Uganda), there was no specific detail on populations with dark skin tones and the associated challenges in diagnosis and prevention.

Pressure injuries have been described as a chronic condition with the majority affecting people living in the community (Jackson et al. 2017). We considered this study necessary to explore the perspectives of community nurses managing pressure injuries in populations with dark skin tones, anticipating that insights gained will contribute to improved strategies for early recognition and prevention of pressure injury in community‐based populations with dark skin tones.

## 3 The Study

### 3.1 Aim

The aim of the study is to examine community nurses' experiences of caring for people with dark skin tones at high risk of developing a pressure injury.

### 3.2 Objective

The objective of the study was to explore and understand the experiences and perspectives of community nurses in caring for individuals with dark skin tones who are at high risk of developing pressure injuries, in order to identify challenges, best practices and areas for improvement in clinical care.

## 4 Methodology

### 4.1 Design

A descriptive qualitative design was used to answer the study aim. The research adhered to the Consolidated Criteria for Reporting Qualitative Research (COREQ) guidelines (Tong, Sainsbury, and Craig 2007).

### 4.2 Study Setting and Recruitment

This study was conducted among registered nurses working in the community who either worked or lived in a region in the South of England. A purposeful sampling method was used to collect rich, relevant and detailed data (Campbell et al. 2020). A call for interviews was sent out to targeted groups via social media which included a link to a Microsoft Forms page with study details and the participant information sheet.

Twenty‐two enquiries were made, and of these, 17 respondents met the inclusion criteria. The first author emailed potential participants to confirm their interest in participating in the interviews. The participants who were willing to take part in the interview were further screened through email to confirm their eligibility. Participants meeting the eligibility criteria were then sent an invitation pack including a consent form and skin tone guide (Ho and Robinson 2015). Recognising that skin tone diversity within clinical practice is a challenging topic (Oozageer Gunowa et al. 2021), participants were offered the choice between a virtual focus group or an individual interview to enhance participation and accessibility.

### 4.3 Inclusion Criteria

Inclusion criteria for the participants were that they (1) were a registered nurse with the Nursing and Midwifery Council (NMC 2024), (2) worked in the community as part of a District Nursing Team, (3) either lived or worked in the specific region in the South of England and (4) able to provide informed consent.

### 4.4 Data Collection

The study interviews were conducted by the first author with participants who provided informed consent. All interviews were digitally recorded with participant consent. The interview guide (Table 1) was developed based on previous studies on skin tone diversity and pressure injuries which focused on data collection within Higher Education Institutions (Oozageer Gunowa et al. 2021). The interviews started with open‐ended questions about their skin tones and their understanding of early visual skin changes and symptoms in pressure injury presentation in a person with dark skin tone. A further question about their views and experiences of seeking early signs of pressure injury was also asked.

**TABLE 1:** Interview Questions.

**Skin Tone** How would you describe your skin tone? Do you feel skin tone and ethnicity are the same thing and should they be used interchangeably? Why? **Pressure Ulcer** 3 What do you understand by the term pressure ulcer? **Community Nursing** 4 What sort of factors do you take into account when making initial skin assessments? 5 How well informed to you feel about redness presenting across the skin tone spectrum? Can you tell me more about this? 6 Do you consider skin tone when carrying out a risk assessment? Why? 7 How do you recognise skin changes? Do you think this is different depending on a person's skin tone? 8 Have you ever provided any leaflets for pressure ulcer prevention? Did they include images of people with dark skin tones? **Nurse Education** 9 Should skin tone variance be recognised in nurse education? Why? How? 10 As a registered nurse have you ever been taught about pressure ulcer identification and people with dark skin tones? 11 In your experience are student nurses/ registered nurses now taught about skin tone variance and the presentation of conditions? Where? 12 What do you feel are the main challenges to the inclusion of skin variation in the nurse education? 13 As a practice supervisor or senior colleague do you talk about skin tone variances when assessing pressure ulcer risk? 14 What knowledge and skills do you feel are important for nurses to best deliver teaching around skin tone variances?

A combination of online focus group interviews (*n* = 3) and individual semi‐structured interviews (*n* = 6) with community registered nurses was conducted to delve deeper into their approaches to assessing early‐stage pressure injuries in individuals with dark skin tones. All community nurses participating in the focus groups and interviews were entered into a prize draw for £125 in shopping vouchers, which was drawn at the end of the study.

### 4.5 Data Analysis

The online interviews, consisting of three focus groups ranging between three and six participants and six individual interviews lasting on average between 30 minutes and 1 hour, were recorded and securely stored per ethics protocols by the first author. Each interview was transcribed verbatim by a professional transcription service for coding. The first author then conducted a thematic analysis of the transcribed data, following the six‐phase framework outlined by Braun and Clarke (2006).

### 4.6 Ethical Consideration

Ethical approval for the study was obtained on the 10 October 2023 from the University of Surrey (FHMS 22‐23239 EGA). Participants were informed that their participation was voluntary and confidential. Both verbal and written consent were obtained from participants before data collection, after they were provided with detailed study information and participant information sheets. Participants were advised of their right to withdraw from the study at any time and without prejudice. However, it was explained that, due to the nature of focus groups, data gathered during these discussions could not be withdrawn after the session had taken place. The sessions were audio‐recorded, with participants informed that the recordings would be transcribed without mentioning their names or any other personal identifiers. Confidentiality has been maintained throughout the study, and all results are presented anonymously.

### 4.7 Rigour and Reflexity

To enhance trustworthiness, this study employed Lincoln and Guba's (1985) criteria, including credibility, transferability, dependability and confirmability of the findings. Two of the three researchers are female registered nurses with doctoral qualifications and extensive experience in both nursing practice and academia and with experience in conducting research into pressure injury. The second author is a male medical doctor working as a research assistant in this study. All researchers had previously worked on studies exploring skin tone bias within healthcare.

Rigour was further ensured by employing several strategies. Accuracy and consistency of the collected data were maintained using automated Microsoft Teams‐generated interview transcripts and audio recordings of interviews. At the end of each interview, the first author reviewed preliminary impressions and asked the interviewees to clarify any discrepancies in understanding. Regular meetings and discussions among authors allowed for ongoing review, reflexivity and the promotion of a comprehensive understanding of the data.

Dependability and confirmability were ensured by maintaining an audit trail, documenting all decisions and steps taken during the research process. Reflexivity was promoted through the researchers' self‐awareness of their potential biases and preconceptions, which were regularly discussed and reflected upon during team meetings. This manuscript adheres to the Consolidated Criteria for Reporting Qualitative Research (COREQ) guidelines (Tong, Sainsbury, and Craig 2007) (see Appendix S1).

## 5 Findings

### 5.1 Characteristics of Participants

The participants, all women, had a range of experience in assessing pressure injuries among people with various skin tones. They reported their own skin tone which ranged from 1 to 6 according to the Ho and Robinson (2015) skin tones tool (Table 2). Using this tool, participants self‐assessed their skin tone by matching it to the closest shade on the upper inside arm, which is divided into six categories to help individuals establish their own baseline skin tone.

**TABLE 2:** Self identified skin tone.

Participant	Skin tone (Ho and Robinson 2015)
NP1	1
NP2	5
NP3	1
NP4	4
NP5	Between 4 and 5
NP6	2
FG1: NP7	1
FG1: NP8	1
FG1: NP9	1
FG1: NP10	1
FG1: NP11	1
FG2: NP12	1
FG2: NP13	5
FG2: NP14	4
FG3: NP15	1
FG3: NP16	1
FG3: NP17	1

**FIGURE 1:**
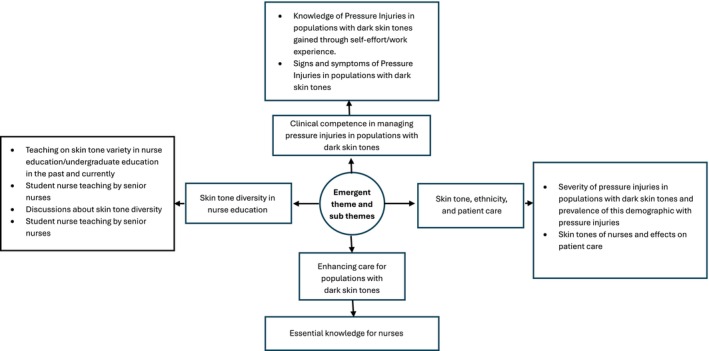
Themes and subthemes.

Following analysis of the interview transcripts, four overarching themes were revealed, and these are as follows: 1. clinical competence in managing pressure injuries in populations with dark skin tones; 2. skin tone, ethnicity and patient care; 3. enhancing care for populations with dark skin tones; and 4. skin tone diversity in nurse education. These themes and associated subthemes are presented below in Figure 1. Subsequent sections of this paper discuss these themes in greater detail with quotes extracted from participant interviews to further expound themes and enrich the narrative.

### 5.2 Theme 1: Clinical Competence in Managing Pressure Injuries in Populations With Dark Skin Tones

This theme and subsequent subthemes highlight the effort put in by nurses to gain knowledge on managing pressure injuries in people with dark skin tones. It also elucidates the signs and symptoms nurses have identified as being associated with pressure injuries and how these presentations differ between skin tones. Furthermore, it highlights how the skin tone of the nurses themselves had an influence on the care provided to patients.

#### 5.2.1 Knowledge on Pressure Injuries in Populations With Dark Skin Tones Gained Through Self‐Effort and Work Experience

Several participants had gained knowledge on pressure injuries in people with dark skin tones through written information, clinical experience, supplemented by the benefit of learning from senior colleagues. One participant stated:

‘We will go and seek out the information [on pressure injuries and people with dark skin tones] ourselves [for example: Best Practice Statement ‐ Addressing skin tone bias in wound care: assessing signs and symptoms in people with dark skin tones] or we use our own…you will use your senior colleagues who you can ask questions…I got my information from…so we have TVNs [Tissue viability nurses] but mostly I would think it's my senior colleagues, like matrons’ (NP5).

The participants noted that meaningful changes to address these issues have only begun to take place in recent years, driven by increased awareness and a growing emphasis on diversity and inclusion in healthcare. Participants in a focus group stated other sources where they got updates on the management of pressure injuries in people with dark skin tones; however, it is important to highlight that these resources were only recently amended if at all following people commenting on how white‐centric the information was:

‘It is usually the National Wound Care Strategy, or the European Advisory Panel for guidance really. And the React To Red campaigns, they tend to have updates on it…we tend to get stuff from the industry as well, they're very good, because obviously they see patients, they do case studies, so we can liaise with them for photos if needed through the reps’ (FG1: NP7).

‘I think there's a Wounds UK conference that I went to, the literature there was the same as what we'd already had what we used at the Legs Matter last year’. (FG1: NP10).

One participant had taught herself about pressure injuries in this population using social media to access scientific publications as soon as possible:

‘Have I ever been taught [about people with dark skin tones and pressure injuries]? No. It's what I've taught myself. So, I'm always reading. And I like the way Twitter enables you to sort of…it kind of helps you keep your finger on the pulse with newly‐published guidance’ (NP3).

#### 5.2.2 Signs and Symptoms of Pressure Injuries in Populations With Dark Skin Tones

The commonest sign of pressure injury identified by nurses working in the community in people with dark skin tones was a change in skin colour. Participants also highlighted the change in skin colour as a notable distinction in the presentation of pressure injuries in people with dark skin tones compared to populations with light skin tones. Pain and a change in skin texture were also highlighted by participants as significant indicators of pressure injuries. A participant stressed the importance of observing skin discolouration and discussed identifying the first stage of pressure injuries saying:

‘You just look for discolouration that is different from the rest of the skin. Because as I said, it's [pressure injuries] harder to identify in darker skin tones… I can't recall a lot of people identifying the first sign of pressure damage for darker skin tone’ (NP4).

Participants acknowledged the challenges and difficulties that can be associated with identifying early signs of pressure damage in people with dark skin tones and noted that early signs can either be missed altogether, or mistaken for something other than pressure damage:

‘I think it's really hard [to assess early‐stage pressure injuries] because…But I think sometimes it [skin changes] can appear maybe more as bruising. It might get mistaken for bruising on darker skin tones. Or people don't…it doesn't click in their head that that's pressure, because it doesn't look like the pictures online of a red or purple discolouration. So, I mean I can only think of two patients that I've ever looked after, with pressure damage, with darker skin tones and yes, it's really tricky, I think’. (FG3: NP17).

The inappropriateness of looking for redness alone when assessing for pressure damage in people with dark skin tones was underscored by participants in a focus group who said:

‘So we're looking at discolouration of the skin… It's very difficult to assess the darker skin tones with redness. Categorising, for example, a category one discolouration, and for example, blanching, erythema, it's more difficult, I think’. (FG3: NP15).

The difficulty in identifying skin reddening in patients with dark skin tones was echoed by other participants. One participant in a focus group when commenting on the difficulty in recognising redness emphasised the need for a more thorough examination and said:

‘Yes, I do think when you're carrying out a pressure area check on someone with a darker skin tone you do have to look a lot more thoroughly, whereas if you've got someone with a lighter skin tone and they've got a red area, obviously it jumps out at you a lot more’ (FG1: NP9).

Participants highlighted the difficulty in recognising pressure injuries in populations with dark skin tones and emphasised the need for more thorough examinations. The need for a meticulous and comprehensive skin assessment was very strongly reflected in the data, as was the need to ensure that nurses are aware of the measures needed to carry out comprehensive skin assessment of people of all skin tones, ‘it is just about making people aware, you have to have a touch of their skin, so you're looking for any warmth and tenderness’. (FG2: NP9).

Participants noted that skin discolouration in people with dark skin tones was not red. One participant in another focus group said:

‘I know people look for red colour, but with my experience with the darker skin, like my skin tone, mostly sometimes it won't be red, it's some sort of discolouration’ (FG2: NP14).

However, participants reported that the language being used was due to their local population demographic stating:

‘where I work it's predominantly English white [population] in most cases, so you hardly meet anyone [with dark skin tone] that's prone to having pressure damage and I guess that's the reason why we stick to the words we use [redness], yes we do mention discolouration, but again, in our heads or in our vision, we're thinking, even myself I'm thinking of white skin, someone with white skin, so yes, I guess, so I'm literally thinking discolouration but I'm referring to a lighter complexion than a dark shade [redness]’ (FG2: NP13).

A participant who noticed that in pressure injuries in populations with dark skin tones, the skin could appear darker saying:

‘If they are a lighter skin tone I'd look for redness, but the darker skin tone the skin will look different the other side and might appear darker’ (NP1).

Conversely, regarding deep tissue injuries, a participant noted that the known deep maroon and purple colours that occur in lighter skinned people might not be as apparent in dark skin tones:

‘I think, as we said before, about the redness and the purple, like if you've got a deep tissue injury, they always say it looks like a deep maroon or purple bruise, but on a darker skin tone, that might not show’. (FG3: NP16).

Participants in the focus groups highlighting pain as a symptom said:

‘But I think a lot of our patients, they become quite painful, so they'll say ‘Oh my bottom's really painful’ (FG3: NP16).

The role of skin palpation in helping make a diagnosis of pressure damage was highlighted by participants who had noticed a change in the texture of the skin:

‘When you touch it, you can maybe feel it a bit raised. Or there's dimpling. So that would also come into it, it feels different… So texture. Because the wound feels different. So you don't necessarily go by sight but you know by texture’ (NP18).

### 5.3 Theme 2: Skin Tone, Ethnicity and Their Influence on Patient Care

This theme explores the interplay between skin tone, ethnicity and their impact on patient care, drawing from insights gathered through in‐depth discussions among community registered nurses. Populations with dark skin tones were found to present with more severe pressure injuries even though such patients were generally fewer on their caseload.

#### 5.3.1 Delayed Identification of Pressure Injuries Among People With Dark Skin Tone

Generally, participants who had managed pressure injuries in patients with dark skin tones noted they had more severe stages compared to participants with light skin tones. This, some participants attributed to missing the early stages of pressure injuries.

Participants in one focus group shared similar experiences and said:

‘By the time we get to them they already have some degree of skin loss (at stage 2), and we miss that early opportunity to prevent further breakdown’ (FG2: NP12).

The nurses reflected on the fact that more could have been done for patients with dark skin tones, recognising that their lack of knowledge and awareness had, in some cases, contributed to inadequate care. This realisation was deeply unsettling for many of them, as they understood the significant impact that timely and accurate pressure injury assessments could have on patient outcomes. A participant in a focus group said:

‘We've looked at a patient [with dark skin tone] who developed skin damage, and it wasn't identified until it was already grade two or three, grade two already there was already breakage, it wasn't grade one. And this is someone that was directly in our care, so it means that there was an issue for identifying’ (FG2: NP13).

Of note was a participant who got a referral when the patient's skin had deteriorated considerably to a more severe stage of pressure injury:

‘Could there have been a point where it wasn't identified that he was experiencing some skin damage, was it missed and now he's got this serious serious wound…I think the wound had deteriorated so much, it was a category four pressure ulcer at that point’ (NP1).

While this patient with the advanced stage of pressure injury had other complications, the participant wondered if something had been missed early and said:

‘With this particular individual it was really hard for the nurses to inspect his skin regularly because of his situation. Nevertheless, nevertheless, was something missed early on?’ (NP1).

When asked the question ‘Do you feel that people with dark skin tone are disadvantaged in a way?’, a participant replied and highlighted the failure in preventing the early stages of pressure injuries in this demographic, meaning that pressure injuries were not identified until Stage 2 or even 3:

‘Definitely. Definitely. Because the fact that we [nurses] fail to even identify from the point where we can prevent it from breaking and that nine out of ten patients, it's when it's already broken’ (NP4).

While patients with dark skin tones who developed pressure injuries were noted by participants to have more severe forms, participants had generally managed few patients with dark skin tones who had developed pressure injuries. Notably, participants with extensive exposure to pressure injuries in this demographic primarily worked in areas with sizable populations of individuals with dark skin.

A participant who reported to have managed pressure injuries in a lot of patients with dark skin tones said: ‘I've seen lots of pressure ulcers in the community in dark skin tone… it's a diverse community’ (NP6).

One participant reflecting on their nursing career expressed their perspective and said:

‘I've had so minimal experience of pressure ulcers, I've done all the training and done all the learning, but I've had very minimal actual face to face pressure experience of people with darker skin tones, almost none [laughs], so I've got all this knowledge but no experience’. (FG1: NP8).

Another participant who worked in an area with a predominantly light‐skinned population expressed worry about the lack of experience saying:

‘In our area, we are a predominantly white area and I think the worry is because we don't get that experience, are we going to miss something in someone with a darker skin tone’. (NP1).

One participant who had managed pressure injuries in a few patients with dark skin tones said:

‘I can only think of two patients that I've ever looked after, with pressure damage, with darker skin tones and yes, it's really tricky, I think’. (FG3: NP16).

#### 5.3.2 Skin Tones of Nurses and Effects on Patient Care

The skin tone of nurses managing pressure injuries in people who had dark skin tones had an effect on the care received by patients as they felt they were better able to identify abnormal skin changes earlier than their lighter skinned colleagues. In response to the question ‘Does it (dark skin tone) make a difference when you're assessing someone's skin?’, a participant replied:

‘It definitely does … So when I look, let's say for example I look on my body, and I've said it's bruised and it's redness, sometimes how it comes up, on some dark skins it looks more purple‐ey. Or I can feel on my skin a certain texture. So texture. Because the wound feels different. So you don't necessarily go by sight but you know by texture’. (NP18).

In response to the question ‘Does having dark skin tone yourself make a difference when you're assessing someone's skin?’ participants indicated that they had increased awareness of the needs of persons with dark skin tones because of their own lived experience.

‘Yes, I would say in a way it does impact the way you care, because you're a bit aware, a little bit aware…but yes, definitely, the fact that I am of a darker skin tone, I guess it makes me a little bit more aware, a little bit more aware. But the fact that it's not emphasised in education and in all the learning and the training, that does impact’ (FG2: NP13).

These participants relied on their own lived experience of having a darker skin tone to assess their patients, as they had not received formal education about skin assessment with people of variant skin tones.

‘I guess you know when you have some kind of damage on your skin, so you identify the skin changes in yourself. So, I guess it makes it easier for you to [assess] on somebody similar. But at the same time, it's much more difficult because as I said, it wasn't something that's included in our training’. (NP4).

It was interesting to note that a participant in a focus group who had a light skin tone assumed nurses with dark skin tones would know what to look out for when examining patients they shared the same skin tone with:

‘And we do have nurses from different ethnic backgrounds as well, so they would then obviously know what to look for in terms of their own skins, so therefore what to look at in terms of patients as well, they know their own skin, therefore would be able to then apply that to other patients as well’. (FG2: NP9).

Conversely, one nurse said

‘I know for a fact that people [all nurses] do struggle, when we have patients, it comes across as if it's something that ‘oh, how am I going to…’, there's a bit of panic because it's not something that is normally talked about’ (NP2).

### 5.4 Theme 3: Skin Tone Diversity in Nurse Education

This theme and correlated subthemes outline the absence of skin tone diversity education for nurses at the undergraduate and postgraduate levels as well as the mentoring practices of senior nurses towards student nurses.

#### 5.4.1 Teaching on Skin Tone Variety in Nurse Education in the Past and Currently

Participants bemoaned the lack of education on skin tone diversity for nurses. With the exception of one nurse, there was a prevailing lack of skin tone diversity education at the undergraduate and postgraduate levels. Of the 17 participants interviewed for this study, only one participant who had a dark skin tone and had been internationally educated received education on skin tone diversity. This participant (from a focus group) remarked:

‘They [academics] did mention about skin tones but they didn't specifically classify it, or they didn't tell us how to do the difference between the darker skin to the whiter skin’ (FG2: NP14).

In the same focus group, other participants echoed similar thoughts on the lack of exposure to undergraduate education on skin tone diversity. It is noteworthy that one participant in the study did not recall the topic of skin tone diversity ever coming up throughout their education journey:

‘It's not something that I've been taught at any level’ (NP6).

Similarly, another participant, reflecting on the undergraduate education said:

‘So when I was an undergrad about 5, 6 years ago, not really. To be honest, I can't remember having any when they were teaching you about redness, erythema, blanching, non‐blanching, they didn't tell you what to look for in a dark skin tone. I can't remember being taught’ (NP2).

The participants noted that meaningful changes to address issues of skin tone diversity have only begun to take place in recent years, driven by increased awareness and a growing emphasis on diversity and inclusion in healthcare. Participants in a focus group who also recounted their undergraduate education lacking training on skin tone diversity said:

‘No, it definitely wasn't, 30 years ago. And it's only recently that we've gone from the React To Red campaign isn't it, that's quite recent, so that just goes to show we weren't doesn't it’. (FG 1: NP8).

However, this participant noted an improvement in current education, saying:

‘I just finished my district nursing training, right, and as part of the physical health assessment there was a presentation on it more in‐depth than before. I'm like okay, this is getting better’ (NP2).

In replying to the question ‘Does anyone really ever talk about skin tone with you at the current present moment, or since you've been employed?’, a participant expressed her frustration stating:

‘My answer is no, my answer is no, no, no, no’ (FG2: NP13).

Notably, a participant with a dark skin tone who was asked if they felt educated on all skin tones replied:

‘I wouldn't say for all skin tones. I would say mainly for the lighter skin tone, yes’ (NP4).

Reflecting on the absence of skin tone diversity in nurse education, one participant said:

‘I think, historically, there's been unconscious bias. I don't think it's ever been thought about. I think it's only… I don't know. To me, it's only sort of been spoken about the last 18 months really. Like looking back now, I just think, I don't know, it's quite unbelievable, isn't it, we live in a multicultural society and we are only just starting to consider it now and there's most probably an element of racism as well in it’. (NP3).

The focus on light skin tones in medical and nursing literature and online images was bemoaned by participants who said:

‘The whole education, the whole anatomy and physiology is around that [people with light skin tones] and everything is around them’. (NP6).

‘But at the moment, pretty much all medical books or whatever you find, it's just the whiter skin tone. There's not really much to do with darker skin’ (NP4).

#### 5.4.2 Student Nurse Teaching by Senior Nurses

It was encouraging to note that some of the participants actively incorporated teaching on skin tone diversity when educating student nurses, while others had never considered this.

In reply to the question ‘Do you talk about it [people with dark skin tones and pressure injuries] now?’, one participant stated:

‘Yes. All the time. All the time. It's in our training. I talk about it on a daily basis and we are really conscious as a team in not just…It's not just around pressure ulcers, it's about all of our tissue viability training, the need to represent that within our training, the darker skin tone. Even things like pictures, making sure that we have pictures of people with wounds with darker skin tones’ (NP1).

Similarly, other participants confirmed teaching nursing students about skin tone diversity, particularly concerning the presentation of pressure injuries but this was contingent on the student, their skin tone and the specific topic being discussed. One participant elucidated:

‘Depending on the student that you're with. Yes [teaching students about dark skin tones and pressure injuries], depending on what sort of student you're with and of what skin tone they are’ (NP6).

Likewise, another participant integrated discussions around skin tone diversity and pressure injuries, stating:

‘So, it's [people with dark skin tones and pressure injuries] just included in what I'm saying if I'm talking about pressure ulcers, then I'll say obviously in the darker skin tone, it will look different, slightly discoloured or maybe just a bit greyish’ (NP4).

Conversely, participants who did not address skin tone diversity with nursing students mainly attributed this to having never considered it, lack of student inquiry, or serving a predominantly white‐skinned population. All participants in one focus group acknowledged their oversight in not teaching students about skin tone diversity:

‘No. I don't think we do, to be honest. I think part of it's probably impacted by the fact that we have a predominantly white patient population. And so I think then, yes, it's not something that I've thought about, to be honest’ (FG3: NP16).

In the same focus group, another participant who had students from different ethnic backgrounds said:

‘I've never once been asked that question and like the other two were just saying, the majority of ours [patients] are from a white background. So obviously I haven't been able to offer that training, because we haven't come across anybody with darker skin tones. And I don't think any of our students have ever questioned that either. I just think it's something we're not thinking about… I suppose if we haven't come across people with different ethnic backgrounds, we can't offer that training’ (FG3: NP17).

During this focus group, the third participant answered in the negative when asked about teaching student nurses. However, they mentioned a recent instance managing pressure injuries in a patient with dark skin:

‘…his very dark skin and multiple pressure injuries that he had, has definitely made an awareness across the whole of the patch, about dark skin and pressure injuries. So it's definitely made us all more aware of it…because we don't seem to have patients with pressure injuries with dark skin, I think it's not been in the forefront of our minds’ (FG3: NP15).

Similarly, the second focus group shared similar sentiments with the initial focus group, with participants simply not thinking about skin diversity when teaching student nurses. One participant attributed this lack of consideration of skin diversity when teaching student nurses to the absence of this topic in their previous education saying:

‘No, I would be lying if I said I did, because it's something I've never thought about, but again it comes down from the teaching that we've had previously at uni, it was never mentioned at all, it was never emphasised or even mentioned’ (FG2: NP13).

#### 5.4.3 Discussions About Skin Tone Diversity

The thought of discussions around skin tone appeared to evoke sentiments of unease, and this was evident in remarks made by certain participants. Participants in one focus group expressed unease and felt the need to discuss skin tones in a sensitive manner. The following ensued between the participants:

‘You need to be well educated that there are differences, and that is just how it is and that it's not someone trying to divide…I don't know how to word it. I hope you'll get what I'm getting at, []. Yes, I think how to do it…’ (FG3: NP16).

‘Without seeming racist, is that what you mean?’ (FG3: NP15).

‘No, I don't mean like that but I mean, kind of. Because you don't want to be like, this is your ‐’ (FG3: NP16).

‘You're different because your skin is different’. (FG3: NP15).

‘Exactly. Exactly that. Without being like that. How to do it in a sensitive, educated manner because you have that knowledge and you know there is scientifically a difference’. (FG3: NP16).

Similarly, another participant said:

‘Maybe when you're handing back over to someone about it and everyone else is about, their understanding is how to deal with a white skin tone rather than dealing with a darker skin tone. So when you're relaying that back to them, can come across as a bit of, ‘oh but, I don't understand, I don't know, because we do this assessment this way, that way’ (NP6).

A participant acknowledged the sensitive nature of discussions pertaining to race and said:

‘I mean, it's always going to be a touchy subject, I think. When you talk about race and stuff like that, it can make a lot of people uncomfortable. But I think in a profession like this [community nursing], we have to overcome that. We deal with uncomfortable situations a lot anyway, so why should this be any different?’ (NP4).

In one focus group, some participants felt comfortable discussing and teaching skin tone differences and said:

‘Yes, because it is no different to people with light skin tones really, the preventative measurements so to speak is exactly the same as what you would put in for a lighter skin tone, the only difference is obviously the subtle changes that you would need to be aware of, but that all comes down to experience, and probably feeling that patient's skin’ (FG1: NP7).

Another participant in this focus group who felt comfortable emphasised the need for tact to avoid coming off as racist:

‘I think personally for me, there is always in the back of your mind that you don't want to come across as being racist in any way, because there is quite a culture out there isn't there of being racist, and it isn't something that you want to be seen as being, because you know you're not’ (FG 1: NP9).

The confidence of internationally educated nurses in practice having discussions about skin tone was also found to be low with one participant suggesting safe places for such discussions:

‘They [internationally educated nurses] definitely haven't got that confidence. But in a safe environment, like our training where there's a small group, you can have those discussions and if you start those discussions then you hope that they will carry on elsewhere’ (NP1).

### 5.5 Theme 4: Enhancing Care for Populations With Dark Skin Tones

To improve care for patients with dark skin tones who developed pressure injuries, participants highlighted topics they thought necessary to gain the knowledge required in the management of patients in this demographic and the importance of research in this area.

#### 5.5.1 Essential Knowledge for Nurses

After discussing the absence of skin tone diversity education generally in nurse education and the teaching of student nurses, participants made suggestions on what knowledge was thought to be essential for nurses to manage pressure injuries in patients with dark skin tones. Participants also highlighted the importance of research involving populations with dark skin tones.

Inclusive education and guidelines were highlighted by some participants who said:

‘Proper education, proper guidelines with the different skin tones, with the different types, so what to do, how to look’ (FG2: NP14).

‘Our education needs to include it and our pressure ulcer competency needs to include it and those competencies need to be achieved’ (NP1).

One participant stressed the critical need for education at all levels of nursing and said:

‘I just feel like more knowledge and education around it is very important and for persons that are above us as well, having that knowledge. Yes. And then they can relay that down to others, so then we can relay it to others, and just keep it moving in that way. But I just feel like more education is needed’ (NP6).

A participant advocated for equal representation for diverse skin tones in nurse education, emphasising the importance of incorporating inclusive education and training:

‘I think more or less have the same information in terms of education and training that they [universities] do for whiter skin tone. Because you can't teach what you don't know. So if that was included, it's mandatory for you to have your pressure ulcer updates’ (NP4).

Similarly, in the context of representing dark skin tones in nurse education, some participants suggested pictures of people with dark skin to represent the stages of pressure injuries:

‘Picture guides, because, again, we know the basics, we know the whats and the hows, it's the actual presentation that we need’ (FG2:NP12).

‘I probably would like some guidance myself, before teaching people, with pictures. Yes, because I would like to know a bit more information myself before teaching people’ (FG3: NP15).

‘I'd like to see some mentioning of what to look for in terms of the skin changes. Not making it generic and it shouldn't be on black and white either – paper – it should be in colour so the image is visible’ (NP4).

The importance of thorough patient examination was highlighted by some participants who said:

‘Do definitely have…feel the area of the wound as well’ (NP5).

‘They [nurses] need to know how to adapt their assessment skills and not just looking for signs, they've got to use all their assessment skills like palpation, changes in skin texture and tone’ (NP3).

A participant specified important questions whose answers would provide important information in caring for patients with dark skin tones:

‘What might a Deep Tissue Injury (DTI) look like. How would you tell the difference between a DTI and a bruise, on someone with darker skin tones. How you could spot the warning signs of a pressure injury. Are they different in someone with darker skin tones?’ (FG3: NP16).

Some participants in a focus group agreed on the importance of making training in skin tone diversity and wound management mandatory for all nurses and said:

‘How to treat general wounds, how to treat pressure wounds, how to treat leg ulcers, as a mandatory training package for all untrained and trained staff in the future… So it really needs to be government enforced, and it needs to be a national standardised training package that everyone should go on’ (FG1: NP7).

Another participant in the focus group who agreed to make the education compulsory said:

‘I agree with [redacted] that actually having compulsory education and training around wound care, pressure ulcer prevention and management etcetera should be compulsory and that would be really beneficial’ (DTS Focus Group_02: F3).

The importance of research involving populations with dark skin tones to establish empirical evidence was emphasised by a participant who said:

‘Because there isn't as much research into it as there are for lighter skin tones. So there does need to be more research and evidence to back up your knowledge and your teaching’. (FG3: NP15).

In a similar vein, another participant said:

‘…the other thing is the importance of engaging people with darker skin tones in this [education], it's really important that researchers do that, too.’ (NP1).

## 6 Discussion

The Queen's Nursing Institute and Royal College of Nursing (2019) recognises community nurses as the backbone of community care delivery, with nurses managing complex care needs all while having the privilege of being a guest in a person's home or in the wider community. Community nurses can truly deliver person‐centred care, recognising the unique needs of individuals as equal partners, often within the comfort of their own homes, all while ensuring patient safety (Coulter and Oldham 2016). However, this study highlights a significant gap in knowledge among nurses working in the community regarding the assessment of pressure injuries among people with dark skin tones. The nurses who participated in the study acknowledged this knowledge gap and many expressed both a sense of shame and guilt.

One area that emerged is missed care. The fact that pressure injuries were more severe in patients with dark skin tones raises concerns about the lack of prevention measures. A patient at risk, regardless of skin tone, and the failure to implement preventive strategies may contribute to more severe injuries. The nurses emphasised the importance of ongoing education and training to ensure that all nurses are equipped with the knowledge and skills necessary to provide equitable care to all patients, regardless of skin tone. This shift towards more inclusive practices is seen as essential for improving patient outcomes and fostering a more just and equitable healthcare system (Nursing and Midwifery Council 2018; Stanford 2020). This study reinforced that education, training and resources both at nursing undergraduate level (Oozageer Gunowa et al. 2021) and within the workplace on this topic were scarce.

Nurses identified that the population demographics of the people they care for influenced their ability to assess individuals with dark skin tones for early signs of pressure damage. Because of this, the language used in practice often focused on redness, which is more easily identifiable in people with light skin tones. Valdez (2021) has highlighted the importance of words and language and how they are used in nursing practice. The use of language that is white‐focused can exclude and alienate students and junior nursing colleagues with dark skin tones (Squire, Gonzalez, and Shayan 2024). These individuals often rely on the knowledge and guidance of senior nurses to shape their practice and care delivery (Eka, Rumerung, and Tahulending 2023). When the language and educational resources predominantly cater to people with light skin tones, it not only marginalises people with dark skin tones but also reinforces a narrow view of what constitutes standard care (Vaismoradi et al. 2022). This exclusion can impede the development of junior nurses, making it difficult for them to provide equitable care and fully understand the diverse needs of their patient population (NHS Race and Health Observatory 2021).

It is essential to adopt more inclusive language and practices that acknowledge and address the unique challenges faced by individuals and populations with dark skin tones, ensuring that all nurses, are well‐equipped to deliver high‐quality, patient‐centred care to all patients and families, regardless of their skin tone (Marjadi et al. 2023). To enable inclusivity and health equity, the language used in clinical practice needs to shift towards more inclusive terms, such as ‘discoloration’, (rather than redness) regardless of population demographics. Several nurses in the study highlighted that this language change is essential for fostering a more inclusive healthcare environment and ensuring equitable care for all patients.

Several nurses interviewed reinforced that pressure ulcers in people with dark skin tones were often detected at a later stage when more extensive damage had occurred (Oozageer Gunowa et al. 2018). This delayed detection may be attributed to difficulties in assessment due to the challenges in recognising early signs on people with dark skin tones, highlighting the increased need for prevention measures. Nurses need to be proactive in preventive care to compensate for assessment difficulties. Interestingly, there was an assumption among nurses with light skin tones that their colleagues with dark skin tones knew what to look for, which was partly true. However, this knowledge was not evidence‐based, and nurses with dark skin tones felt that their assessments were based on personal experience rather than scientific evidence. This often puts them in an uncomfortable position when advising colleagues. Furthermore, it is important to recognise that relying on nurses with dark skin tones can place an undue burden on them and allow nurses with light skin tones to evade their own duty of care to patients on the basis of skin tone. Additionally, there may be an underlying bias for some nurses that people with dark skin tones are less prone to breakdown, which could contribute to a lack of presentation and delayed diagnoses in those with dark skin tones. Addressing this misconception is crucial to ensure timely and appropriate care. It is essential for allies to actively participate and take responsibility to deliver inclusive and anti‐discriminatory care (Thorne 2022).

Similar to findings in other studies (Oozageer Gunowa et al. 2021; Wounds 2021), the nurses reported feeling uncomfortable discussing or considering skin tone diversity, fearing they might be perceived as racist. This discomfort was often rooted in a lack of confidence and experience in addressing issues of skin tone and diversity openly. As a result, nurses were inadvertently prioritising their own comfort over the delivery of truly person‐centred care, which is fundamental to both patient safety and health equity (Rossiter, Levett‐Jones, and Pich 2020; Edgman‐Levitan and Schoenbaum 2021). The reluctance to engage in conversations about skin tone diversity can lead to significant gaps in care, particularly for patients with dark skin tones who may have unique clinical presentations and care needs (Oozageer Gunowa, Oti, and Jackson 2024). By avoiding these discussions, nurses may miss critical signs of pressure injuries and other conditions that present differently in people with dark skin tones, thus compromising patient outcomes (Julka‐Anderson et al. 2024; NHS Health and Race Observatory 2023). Furthermore, this avoidance perpetuates a cycle of inequality and reinforces systemic biases within healthcare settings.

In summary, this study underscores the need for improved education and resources on assessing pressure injuries in people with dark skin tones. It highlights the importance of using inclusive language and addressing the discomfort that nurses may feel in discussing skin tone diversity, to ensure equitable and effective patient care.

### 6.1 Strengths and Limitations

The participants in this study varied widely in terms of age, years of experience, skin tone and the frequency with which they cared for individuals with dark skin who were either at risk of developing pressure injuries or were in the early stages of pressure injuries. This diversity among participants provided a broad spectrum of insights and experiences, enriching the study's findings. Many participants were likely drawn to the study through advertisements posted in professional networks on social media.

However, a limitation of the study was the introduction of self‐selection bias, as participants volunteered themselves. Those who volunteered might have had a particular interest or personal connection to the topic, potentially influencing their responses and perspectives. Most of the participants had a light skin tone. This highlights the need for further research exploring how nurses with different skin tones perceive and approach the care of patients with dark skin tones, to better understand the nuances and challenges in providing equitable care.

### 6.2 Recommendations for Further Research

Further research should explore the importance of patient‐centred care that respects and responds to the unique needs of individuals with dark skin tones. This includes actively listening to patients' experiences and symptoms across various settings and internationally to inform and shape current and future practices around patient safety. Additionally, it is fundamental to identify and analyse the barriers to implementing inclusive practices and guidelines in clinical settings through dedicated research.

### 6.3 Implications for Policy and Practice

The study offered a voice to community nurses to express their experience of caring for people with dark skin tone at a high risk of developing a pressure injury. This insight highlights the need to inform training programmes both at undergraduate and postgraduate level which focus on the management of pressure injuries in people with dark skin tones. Policies should mandate the inclusion of skin tone diversity in nursing curricula and ongoing professional development. Clinical guidelines and protocols should be revised to ensure they are inclusive of all skin tones. This includes the use of appropriate language and imagery that reflects the diversity of the patient population.

## 7 Conclusion

This study has highlighted gaps in knowledge about pressure injuries in persons with dark skin tones and adds to the literature identifying gaps in nurse education about diversity of skin tones and its effects on early recognition of preventable patient harm such as pressure injury. There is also evidence of assumptions made that nurses who themselves have dark skin tones will somehow recognise and respond to early pressure damage. This study has highlighted the need to have open discussion about diversity in skin tone, and the need for these discussions to occur across the spectrum of clinical, research and educational contexts of nursing. Only through open discussion and inclusion of people of all skin tones in research and education will nursing truly achieve its obligations to provide equity of care for all persons, regardless of skin tone.

## Conflicts of Interest

The authors declare no conflicts of interest.

## Peer Review

The peer review history for this article is available at https://www.webofscience.com/api/gateway/wos/peer‐review/10.1111/jan.16533.

## Supporting information


Appendix S1.

## Data Availability

The data that support the findings of this study are available on request from the corresponding author. The data are not publicly available due to privacy or ethical restrictions.
